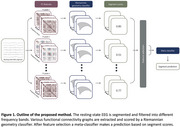# Decoding Dementia: Classifying Alzheimer's and Frontotemporal Dementia with EEG and Riemannian Geometry

**DOI:** 10.1002/alz70856_105377

**Published:** 2026-01-08

**Authors:** Arne Van Den Kerchove, Tjaša Mlinarič, Barbara Verovnik, Zoe Isabella Barinaga, Marc Van Hulle

**Affiliations:** ^1^ KU Leuven, Leuven, Belgium; ^2^ University of Ljubljana, Ljubljana, Slovenia

## Abstract

**Background:**

Frontotemporal dementia (FTD) is frequently misdiagnosed as Alzheimer's disease (AD) due to their overlapping symptoms, leading to a decreased quality of life and misallocation of resources. Research indicates that neuroimaging techniques outperform cognitive tests in differentiating between these conditions, with electroencephalography (EEG) offering a cost‐effective, accessible, and faster alternative. Moreover, resting‐state EEG is less taxing on patients, which is particularly important for those with cognitive impairments.

While EEG‐based classification between AD and healthy controls (HC), as well as FTD and HC, has shown promising results, accurately distinguishing AD from FTD remains challenging. Functional connectivity (FC) graphs are key features used to construct machine learning (ML) models for this problem. However, most of the research using ML with FC features has been conducted using flat Euclidean metrics, even though some connectivity graphs naturally reside on non‐Euclidean manifolds. Riemannian geometry provides a more suitable framework for analysing and classifying complex data structures, like some FC graphs, enhancing ML performance.

**Method:**

We propose a classification strategy using Riemannian geometry and resting‐state EEG to differentiate between AD, FTD, and HC (Figure 1). This method utilizes stacked generalization to combine predictions obtained by a set of Riemannian geometry classifiers each trained on one of various FC features across different frequency bands (delta, theta, alpha, beta, gamma). A logistic regression meta‐classifier combined with sequential feature selection is used to reach a decision based on the predictions per FC feature.

**Result:**

Our proposed model was evaluated on an openly available dataset (doi:10.18112/openneuro.ds004504.v1.0.7) with 36 AD, 23 FTD, and 29 HC age‐matched subjects. Leave‐one‐subject‐out cross validation yielded the following classification performances for 3 binary classification problems while accounting for class imbalance: AD/FTD: 74%, AD/HC: 85%, FTD/HC: 74%.

**Conclusion:**

By using smaller, efficient models that account for data geometry and a meta‐classifier, our proposed method reaches good performance while allowing for enhancement through alternative feature selection and hyperparameter optimization. Additionally, our method identifies the most informative FC graphs for classification, which are valuable for model interpretation and can provide specific distinctive neural signals that can be used in future research to develop diagnostic tools.